# Saccadic movements during an exploratory visual search task in patients with glaucomatous visual field loss

**DOI:** 10.5935/0004-2749.2022-0366

**Published:** 2024-03-05

**Authors:** Mirella Almeida Oliveira, Cassia Senger, Raquel Pantojo Souza, Carlos Gustavo de Moraes, André Messias, Jayter Silva Paula

**Affiliations:** 1 Department of Ophthalmology, Otorhinolaryngology and Head Neck Surgery, Faculdade de Medicina de Ribeirão Preto, Universidade de São Paulo, Ribeirão Preto, SP, Brazil; 2 Department of Physics, Faculdade de Filosofia, Ciências e Letras de Ribeirão Preto, Universidade São Paulo, Ribeirão Preto, SP, Brazil; 3 Bernard and Shirlee Brown Glaucoma Research Laboratory, Edward S. Harkness Eye Institute, Department of Ophthalmology, Columbia University Medical Center, New York, NY, USA

**Keywords:** Glaucoma, open angle, Saccades, Eye movements, Visual fields, Vision disorders

## Abstract

**Purpose:**

To evaluate the saccadic movements of patients with visual field loss due to primary open-angle glaucoma.

**Methods:**

Thirteen patients with good visual acuity (0.2 logMAR or better) (seven patients with primary open-angle glaucoma 65 ± 13 years) and six controls (51 ± 6 years) yielded a comprehensive ophthalmological examination, including Humphrey Visual Field tests (SITA-Standard 24-2), and performed a monocular, exploratory digital visual search task that quantifies the duration for finding the number “4” on a random array of digits distributed on the screen. After individual adjustments of the angle and distance positioning, the screen was spatially matched with the 24-2 visual field, and divided into five areas for analysis. During the task, saccades were simultaneously recorded in the same eye with a video-based eye tracker.

**Results:**

The patients with primary open-angle glaucoma showed a significantly higher number of saccades/screen (median ± interquartile range, 59.00 ± 29.00 *vs*. 32.50 ± 19.75 saccades (p=0.027) and visual search time per screen (38.50 ± 60.14 *vs*. 23.75 ± 8.90 seconds (p=0.035) than the controls did. Although the univariate analysis indicated a significant correlation with visual field mean deviation (coefficient=26.19 (p=0.02), only the visual search time/screen was significantly associated with the number of saccades/screen in the multivariate regression model (coefficient=0.55 (p<0.001). Overall, no significant correlation was observed between the sectorial number of saccades and the sensitivity of the five visual field areas.

**Conclusions:**

The patients with primary open-angle glaucoma show impaired search performance and showed a higher number of saccades needed to find stimuli when performing the exploratory visual task.

## INTRODUCTION

Glaucoma may be considered as a chronic optic neuropathy that is mostly associated with peripheral visual field (VF) loss, since it preserves a good visual acuity for a significant period of time in many patients^(1,2)^. However, patients with glaucoma tend to have a decreased quality of life which has been associated with wide-ranging changes in VF^(3)^. Moreover, glaucomatous changes in VF have been associated with difficulties in various daily activities, such as reading, walking, and driving^(4,5,6)^. However, in patients with glaucoma, several activities dependent on peripheral vision might not be influenced by VF changes. Thus, it is important to determine the other factors involved in different visual search (VS) processes in a given environment or setting^(2)^. During a VS task, peripheral vision would enable coarse-scale encoding which serves as a signal for saccadic eye movements, allowing the placement of the searching target in the foveal region aiming a high-resolution evaluation^(7,8,9)^. Several parameters including visual acuity, VF, and contrast sensitivity have been associated with VS performance and the coordination of saccades. Conditions related to central scotomas and peripheral VF defects may impact VS tasks^(10,11,12,13)^.

Several studies have shown that patients with glaucoma experience difficulties when undertaking VS tasks, including the topographic association between worse VS performances and peripheral VF losses in patients with primary open-angle glaucoma (POAG)^(14,15,16,17,18)^. Other compensatory mechanisms have been implied in this process, such as saccades^(2)^. Those mechanisms could positively influence daily tasks involving VS in patients with peripheral VF loss, but such relationship has not been topographically evaluated.

Since VS performance may be correlated with saccades and peripheral VF losses as observed in glaucoma patients, we investigated the topographic association between the distribution of saccades and peripheral VF defects during an exploratory VS digit-based task in normal central vision patients with POAG and healthy controls.

## METHODS

### Participants

This study was approved by the local ethics committee (protocol number 660.663/2014), and all subjects provided informed consent prior to their participation. The sample size was calculated using the results regarding VS performance as described by Senger et al.^(17)^, assuming a test power of 80%, an alpha of 0.05, and a rate of data losses of 10%.

Initially, 57 eyes from 29 POAG patients and 28 healthy individuals (controls) were selected from the ophthalmology outpatient clinic of the Clinical Hospital of the Ribeirão Preto Medical School, University of São Paulo, Brazil. The inclusion criteria were as follows: aged 40-80 years, good cognitive skills, availability to attend all appointments, and ability to perform all examinations following the protocol. On the other hand, the ocular inclusion criteria were as follows: no optical media opacities, refractive error of ≤±6D spherical and ≤3D cylindrical, best corrected visual acuity (BCVA) better than 0.2logMAR, and a sufficient ability to operate the mouse and complete understanding of the VS task (considering the finalization of tasks in less than 15 minutes, keeping the initial head position). The exclusion criteria were as follows: uncontrolled systemic comorbidities, previous eye surgeries (except trabeculectomy or phacoemulsification performed more than 12 months ago), ocular diseases that could interfere with visual function assessments, and unreliable results on standard automated perimetry (SAP) (e.g., fixation losses, false- positive or false-negative results greater than 20%, 30%, and 30%, respectively).

The diagnosis of POAG was confirmed previously by a chart review of patients with at least three measurements of intraocular pressure (IOP) higher than 21 mmHg as performed on different days using a Goldmann tonometer, with or without antiglaucoma medication: open angles at gonioscopy; cup-to-disc (CD) ratio of ≥0,6 or any localized signs of glaucomatous optic neuropathy; and suggestive glaucomatous VF observed in the SAP based on the criteria proposed by Hodapp-Parrish-Anderson^(19)^.

All participants were assessed by the same examiner (CS) in the following sequence: (1) comprehensive ophthalmological examination (BCVA, ocular motility, refraction, biomicroscopy, tonometry, and gonioscopy); (2) VF examination (SAP strategy 24-2, SITA-Standard; Humphrey Visual Field Analyzer 750-i, Carl Zeiss, Dublin, CA, EUA); (3) spectral domain optical coherence tomography (SD-OCT) (Spectralis OCT, Heidelberg Engineering, Germany); and (4) exploratory visual search task.

### Exploratory visual search task

Each participant sat comfortably in front of the computer screen (Samsung monitor, UN32FH4205), with their face positioned on the chin rest, the right eye occluded, and the left eye corrected for near vision. Moreover, the left eye was aligned with a central stimulus which was presented on the initial screen before the test started. A fixed distance of 62 cm was always maintained between the center of the screen and the left eye.

The custom software was created using Borland Delphi 7.0 which displayed a random arrangement of digits (0-9, in Arial font, size 14) on a 70cm x 40cm monitor that were distributed in regions which scotomas were equally as likely to be present as they were to be absent. The patients moved the mouse with their dominant hand in the direction of the predefined digit (digit “4”). Each trial had at least one digit “4”, randomly distributed in location and number per screen as previously described^(17)^. They marked this target by hovering the cursor over it. The program then changed the color of the target digit to red, preventing duplicate searches for the same element. After finding all digits of “4”, the screen was cleared automatically, and the next test was initiated by clicking a button in the center of the screen again. This ensured the initial central fixation. Thus, after finding all the targets for a given screen, a new screen was automatically restarted and the subject progressed to the next phase of the test, aiming and clicking the central button successively until 10 screens had been completed.

### Eye tracking

Each participants’ eye movement was recorded during the VS task using a head-mounted monocular eye tracker that tracks the movement of the pupil and corneal reflection eye landmarks at a 60 Hz rate. This device was attached to a video camera fixed on the patient’s frontal region (ISCAN ETL-200, Head-mounted Eye Tracking Laboratory, Iscan Inc., MA, USA).

The infrared camera array has 256 vertical x 512 horizontal pixels, and the pupil center coordinates are calculated to represent eye position. Considering a total screen size of 40cm vertical x 70cm horizontal, the spatial resolution can be estimated in 0.14cm horizontally x 0.16cm vertically. The ISCAN ETL-200 was calibrated prior to each test, asking subjects to direct their gaze toward four marks positioned on the screen corners. A built-in calibration procedure was used to convert pupil position data to degrees of visual angle. Blinks were also detected and filtered using an integrated ETL-200 feature. The detection algorithm continuously compares the incoming real-time pupil extent information to a template of the eye with the pupil fully visible, which is initially set by the operator. It then registers and counts the number of blinks based on a percentage of the eye closure parameter. During calibration, the operator ensures that the four corners were within the visible area in which eye movements could be tracked without losing the pupil area underneath the lateral or nasal cantus of the eyes. But even if the subjects will look outside that area, the system will detect this movement as a blink, and the data would be deleted.

**Figure 1:**
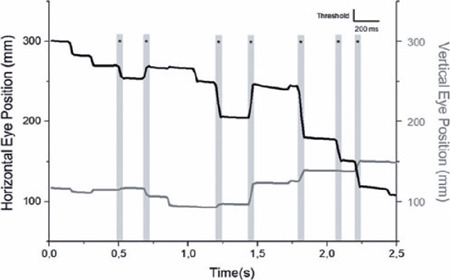
Samples of eye movements from Patient #4 (POAG group) during 2.5 seconds of the VS task. Observe the trace of the sequence of eye movements generated by the Matlab program demonstrating both the vertical (gray line) and horizontal eye movements (black line). Gray shadow columns with an asterisk in the graph represent the detection of saccadic movements.

A custom program was created using MATLAB (MATLAB and Statistics Toolbox Release 2016b, The MathWorks, Inc., Natick, Massachusetts, USA) to define and analyze saccades and fixations. A saccade was defined as an abrupt change in the eye position larger than 9% of the eye movement total range in any direction and should be either preceded or succeeded by a fixation that is defined as the absence of saccades for a time interval greater than 200ms (Figure 1).

**Figure 2:**
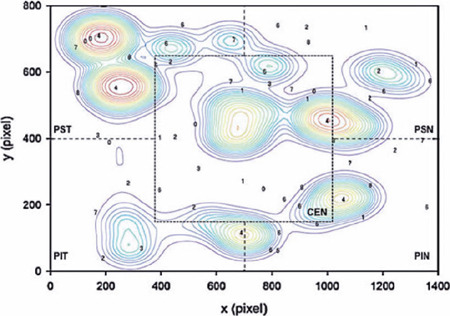
Schematic diagram of the screen results of a healthy subject (control group) with the topographical division of areas, representing the five visual field sectors proposed in the protocol. Colored polar plots represent areas with a high frequency of saccades detected on the screen, mostly concentric to the targets. The quantile density estimation divides the screen into colored contours that represent quantile areas of 10% of frequency. Note the location of five targets randomly distributed during this test (number “4”), which presented with increased saccadic movements.

A topographic correspondence was established between localized VS performance and the VF sensitivity results of the five sectors divided from the APP printouts using trigonometric relationships and following the same protocol described by Senger et al.^(17)^ (Figure 2).

PST= peripheral superior temporal; PIT= peripheral inferior temporal; PSN= peripheral superior nasal; PIN= peripheral inferior nasal; CEN= central.

Variables, such as the location of the digits found on the screen (x and y coordinates in pixels), VS time (time required to find all targets), and the number of saccades, were obtained for all screens and stored in a database for analysis. Furthermore, the program “DQW Data Acquisition & Control Software” recorded and archived the vector data of the saccades.

Data from all participants in both groups were analyzed, and sex, age (years), BCVA (logMAR), IOP (mmHg), CD ratio, peripapillary nerve fiber layer thickness (RNFL, µm), mean deviation (MD) of VF sensitivity (in dB), VS time per screen (VS time/screen, seconds), and the number of saccades/screen were compared.

### Statistical analysis

The data collected were described using proportions for categorical variables and median and interquartile range (IQR) for continuous variables. Given the nature of the data, the groups were compared using non-parametric tests (Mann-Whitney U test) for continuous variables, complemented by tests using contingency tables for categorical data (software Prism 6.0, GraphPad Software Inc., CA, USA).

The examination data obtained were considered reliable when at least four screens presented no technical issues and an exact time alignment for each participant’s trial. Moreover, multivariate regression with mixed effects models was used to determine a possible correlation between the sectorial number of saccades/ screen and each of the five sectors of the VF. Possible relationships between the number of saccades/screen and the variables MD, VS time/screen, BCVA, glaucoma diagnosis, age, and sex were evaluated through univariate and multivariate regression analyses (Stata 14.2, Stata Corp., Texas, USA). Furthermore, p-values of <0.05 were considered statistically significant.

## RESULTS

**Figure 3:**
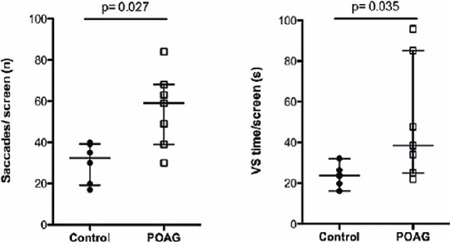
Comparison of the number of saccadic movements per screen (saccades/screen, left) and the VS time per screen (VS time/screen, right) between patients with glaucomatous visual feld defect and good visual acuity (POAG) and healthy controls, as recorded during the VS task. Error bars display medians with IQR.

The POAG group did not differ from the controls in terms of sex, age, BCVA, and IOP (13 participants: 7 POAG, 64.0 ± 18.5 years; 6 controls, 55.0 ± 8.0 years). However, this group had a higher CD ratio (0.80 ± 0.20 *vs.* 0.30 ± 0.08; p=0.002) and lower RNFL values (76.00 ± 26.50 *vs.*103.00 ± 15.05 µm); p=0.003) than the controls (Table 1). The POAG group also showed a significantly higher number of saccades/screen (59.00 ± 29.00 vs. 32.50 ± 19.75 saccades, p=0.027; U=5.00) and VS time per screen (38.50 ± 60.14 vs. 23.75 ± 8.90 seconds, p=0.035; U=6.00) (Figure 3) than the controls did. Table 1 shows the results of the other features compared between the groups.

**Table 1:** Demographic data, general conditions, and clinical characteristics of all 13 subjects analyzed in relation to saccadic behaviors.

Features	POAG	Controls	p-value
Sex (M: F)	3:4	2:4	1.000
Age (years)	64.00 ± 18.05	55.00 ± 8.00	0.384
BCVA (logMAR)	0.10 ± 0.15	0.00 ± 0.10	0.764
IOP (mmHg)	15.00 ± 3.50	13.50 ± 2.75	0.888
CD ratio	0.80 ± 0.20	0.30 ± 0.08	0.002
Thickness RNFL (µm)	76.00 ± 26.50	103.0 ± 15.05	0.003
VS time/screen (s)	38.50 ± 60.14	23.75 ± 8.90	0.035
VS time/stimulus (s)	4.00 ± 3.00	2.00 ± 1.5	0.038
Saccades/screen (n)	59.00 ± 29.00	32.50 ± 19.75	0.027
Saccades/stimulus (n)	8.42 ± 5.48	6.76 ± 3.86	0.295
Saccades size (mm)	152.40 ± 53.30	140.30 ± 57.6	0.628
Fixation time (s)	0.33 ± 0.11	0.32 ± 0.05	0.628
Screens (n)	7.00 ± 5.00	5.00 ± 3.25	0.940
Stimuli (n)	35.00 ± 29.00	22.00 ± 23.75	0.721

M:F= male:female. The other results are described in mean ± SD values. BCVA= best corrected visual acuity. IOP= intraocular pressure. CD= cup-to-disc. RNFL= retinal nerve fiber layer. VS= visual search. Data are presented as median ± interquartile range, excepting for “sex.” The Mann-Whitney U test was used for all comparisons except for “sex,” which was compared using Fisher’s exact test.

Although the univariate analysis showed a significant correlation with MD (coefficient=-26.19, p=0.02), only VS time/screen was significantly associated with the number of saccades/screen in the multivariate regression model (coefficient=0.55, p<0.001) (Table 2). Nevertheless, the multivariate regression analyses performed separately by groups showed that only patients with POAG presented a significant correlation between saccades/screen and both VF MD (coefficient=-18.90, p=0.037) and VS time/screen (coefficient=0.59, p=0.002) (Table 3). Furthermore, it is important to consider that no significant association was observed between the sectorial number of saccades and the sensitivity of the five VF areas.

**Table 2:** Univariate and multivariate regression analyses between the number of saccades/screen and age, sex, BCVA, VF MD, and VS time/screen

Variable	Univariate		Multivariate	
	Coef.†	p-value	Coef.†	p-value
Age (58.0 ± 16.5 years)	4.34	0.221	-	-
Sex (5 F/ 8 M)	65.65	0.475	-	-
BCVA (0.0 ± 0.1 logMAR)	-470.2	0.200	-	-
MD (-2.03 ± 6.39 dB)	-26.19	0.020	-12,41	0.064
VS time/screen (26.40 ± 20.35 s)	0.65	<0.001	0.55	<0.001

BCVA= best corrected visual acuity; MD= visual field mean deviation; VS= visual search; †= correlation coefficient. Data in parentheses are presented as median ± interquartile range.

**Table 3:** Multivariate regression analysis between the number of saccades/screen and the visual feld MD and VS time/screen by group (POAG, n=7; controls, n=6)

Variable	POAG			Controls		
	Median ± IQR	Coef.†	p-value	Median ± IQR	Coef.†	p-value
MD (dB)	-6.64 ± 5.75	**-18.90**	**0.037**	-0.89 ± 1.71	-27.72	0.561
VS time/screen(s)	38.50 ± 40.14	**0.59**	**0.002**	23.75 ± 8.90	-0.79	0.350

POAG= primary open-angle glaucoma; MD= mean deviation (from visual field); VS= visual search; IQR= interquartile range; †= correlation coefficient.

## DISCUSSION

Many daily tasks depend on an accurate visual system that is capable of seeking targets in complex scenes or environments, which may be altered in patients with glaucoma^(15,16,17,20,21,22)^. Overall, patients with glaucoma and peripheral VF defects performed worse in the VS task in the present study. We observed that the POAG group showed an increase in the VS time/screen and a greater number of saccades/screen than the controls did. Consistent with previous studies, the findings of the present study suggest that VF loss may yield limitations in the VS process along with changes in eye movements^(15,16,18,20,21,22,23,24,25,26)^.

In this context, Crabb et. al. assessed saccades during tasks of perceiving danger in driving scenes and found that glaucomatous patients showed a higher number of saccades per second^(16)^. Furthermore, patients with glaucoma showed a prolonged saccade reaction time but no differences in several saccades parameters compared with the healthy controls^(17)^. Similarly, we identified only a significant increase in the number of saccades among patients with glaucoma. This increased number of eye movements performed during search suggests the existence of compensatory mechanisms deployed to overcome visual impairment, such as peripheral VF loss^(16,18,23,25,27)^.

Interestingly, this visual compensation might vary according to the task. For several static and dynamic visual stimuli, the number of eye movements increase^(10,16,21,25)^, but a decrease has also been observed in patients with glaucoma during a VS task of everyday scenes as displayed on a computer screen^(20)^. In cases with lower visual acuity or deeper visual field loss, such divergences could be explained by the differences between tasks and also by the magnitude of visual loss^(22,24)^.

The VS performance depends on both central and peripheral vision in most of daily activities. During a VS task, stimuli present in the periphery can determine eye movement behaviors^(15,16,20,23,28)^. The increase in the VS time of patients with glaucomatous VF losses is certainly due to an inability to use information presented in the peripheral region with a VF defect^(15,16,22,26)^.

Previous studies have shown a direct association between poor performance in the VS task and predefined areas of glaucomatous VF loss^(17)^. The main objective of the present study is to evaluate the topographic relationship between areas with VF loss and the saccade spatial distribution. However, no significant relationship was observed. Due to the nature of the exploratory and sequential VS task, all eye movements would likely change the relative position of the scotoma areas on the screen with the stimuli, hindering a topographic correlation analysis, and this particular cohort showed only localized VF losses which may help explain our results. Patients with larger scotoma or more diffuse VF loss could have yielded different results. A potential topographical association in eyes with worse VF defects is speculative since we evaluated a small number of patients who presented exclusively mild to moderate VF losses (MD better than -12 dB).

Furthermore, searching is a more complex visual task than visual acuity (discrimination on the central visual field) or VF measurement (light stimulus perception on the visual peripheric field), because the visual system must perform target discrimination on the visual periphery and provide information for performing the gaze (locating and fixating the targets), motor system (to realize the eye movements), limbs (to perform the task), and a central system (cognition) that plans the overall sequence of actions^(29)^. In this context, since a diminished integration of visual information and cerebellar function has been observed in glaucoma patients, this idea could at least partially explain a possible lack of correspondence between the areas of VF losses and the search direction^(30)^.

Despite the attempt to mitigate ocular and cognitive cofounding factors, as well as potential shortcomings related to the sequential characteristics of this task, our study still has several limitations. We used a stimulus generated on a computer screen that can be randomized and standardized and roughly mimic important daily visual tasks. Although the test is applied with uniform exposure to the target, including size, contrast, location, exposure time, and output of spatial information, it requires the use of a computer mouse, which needs a preserved motor coordination. Furthermore, the evaluation of eye movements was performed in only one eye which likely limits the reproduction of real-life experiences. For future studies, the development of a new research platform which does not depend on manual motor coordination and enables binocular assessment would be advantageous.

In the present study, we observed an increase in the number of saccades per screen as well as an increase in VS time in patients with POAG who presented with localized VF loss and normal visual acuity, suggesting an impaired search strategy. The lack of topographical correspondence between VF loss and search direction and the absence of correlation between total VF loss and the number of saccades indicate that searching should be investigated in glaucoma as a tool to improve the estimation and understanding of the impact of the disease on patients’ daily life visual tasks.
